# Seismological constraints on the crustal structures generated by continental rejuvenation in northeastern China

**DOI:** 10.1038/srep14995

**Published:** 2015-10-07

**Authors:** Tian-Yu Zheng, Yu-Mei He, Jin-Hui Yang, Liang Zhao

**Affiliations:** 1 Key Laboratory of Earth and Planetary Physics, Institute of Geology and Geophysics, Chinese Academy of Sciences, Beijing, 100029, China; 2 State Key Laboratory of Lithospheric Evolution, Institute of Geology and Geophysics, Chinese Academy of Sciences, Beijing, 100029, China

## Abstract

Crustal rejuvenation is a key process that has shaped the characteristics of current continental structures and components in tectonic active continental regions. Geological and geochemical observations have provided insights into crustal rejuvenation, although the crustal structural fabrics have not been well constrained. Here, we present a seismic image across the North China Craton (NCC) and Central Asian Orogenic Belt (CAOB) using a velocity structure imaging technique for receiver functions from a dense array. The crustal evolution of the eastern NCC was delineated during the Mesozoic by a dominant low seismic wave velocity with velocity inversion, a relatively shallow Moho discontinuity, and a Moho offset beneath the Tanlu Fault Zone. The imaged structures and geochemical evidence, including changes in the components and ages of continental crusts and significant continental crustal growth during the Mesozoic, provide insight into the rejuvenation processes of the evolving crust in the eastern NCC caused by structural, magmatic and metamorphic processes in an extensional setting. The fossil structural fabric of the convergent boundary in the eastern CAOB indicates that the back-arc action of the Paleo-Pacific Plate subduction did not reach the hinterland of Asia.

Continents generally contain Precambrian nuclei enclosed by later tectonic domains and are repeatedly modified by structural, magmatic and metamorphic processes after their formation. The pre-existing continental crust is partly rejuvenated by these tectonic processes and forms new structural fabrics and juvenile crust^1^,^2^. Crustal rejuvenation in a tectonic active continental region is a key process in the evolution of continental crusts and has become an important issue associated with the formation of economic resources and geological hazards. In the Aegean region of the Mediterranean, the active rifting, formation of metamorphic core complexes (MCCs) and exhumation of high-pressure and low-temperature metamorphic rocks in a lithospheric extensional setting related to slab roll-back have acted to rejuvenate the continental crust^3^,^4^. In western North America, late Mesozoic and Cenozoic tectonism was largely controlled by the eastward progressing subduction of the Farallon Plate^5^. The structural and compositional evolution of the continental crust and lithosphere in the back-arc tectonic setting in North America has become an advanced research hotspot^6^,^7^,^8^,^9^,^10^. A number of studies have shown that crustal rejuvenation is a key issue shaping the characteristics of present continental structures and components in continental margins adjacent to oceanic plates. Crustal rejuvenation has been delineated by geological and geochemical observations. However, crustal structures have not been well constrained, which is mainly because pertinent observations are scarce.

Northeast China experienced strong lithospheric extension, deformation and magmatism during the amalgamation of the Siberia Craton and North China Craton (NCC) and the subduction of the Paleo-Pacific Plate, which has occurred since the Mesozoic. In particular, the eastern NCC, which is a typical region in East Asia, has experienced lithospheric removal, with the thick, refractory cratonic lithospheric mantle replaced by a thin, fertile lithospheric mantle, and this process is associated with the strong crustal extension, magmatism and Cu-Au-Mo-Fe mineralization in the Late Mesozoic to Cenozoic^11^. The shallow geological records of the granitic magmatism, lower crustal xenoliths and MCCs in the NCC indicate the modification of continental crustal structures and components. Here, we reveal the details of the crustal structure obtained from a seismic profile across the NCC and Central Asian Orogenic Belt (CAOB). The unusual crustal fabrics provide strong constraints and important information regarding crustal transformations in a back-arc tectonic setting.

## Geologic setting

**Figure 1:**
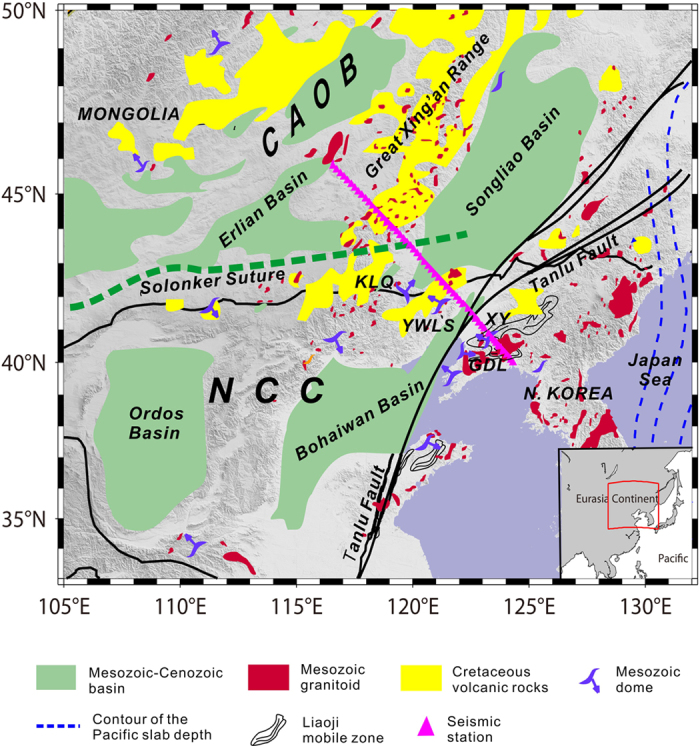
Simplified tectonic map of the study region showing the location of the NCISP-6 seismic array. The magenta triangles represent the seismic stations. CAOB—Central Asian Orogenic Belt, NCC—North China Craton, GDL—Gudaoling metamorphic core complexes (MCC), KLQ—Kalaqin magmatic dome, XY—Xiuyan magmatic dome, and YWLS—Yiwulushan MCC. The map was created using the generic mapping tool (GMT) software included with the ETOPO2 model^42^.

Northeast China is contains the northeastern portion of the NCC and the eastern portion of the CAOB, and the Soloker suture acts as a convergent boundary (Fig. 1). The CAOB is a giant accretionary orogen between the Siberian Craton and the NCC and Tarim Craton. This orogen was formed by the subduction of the Paleo-Asian Ocean plate, the amalgamation of different types of terranes and the accretion of juvenile materials during the Late Permian to Early Triassic^12^. The eastern CAOB experienced intensive extensional deformation and magmatism in the Late Jurassic and Early Cretaceous^13^. Widespread granitic rocks are exposed and constitute the eastern part of a huge granitic belt in the CAOB^14^. Late Mesozoic volcanism was widespread throughout northeastern China and primarily clustered in the Great Xing’an Range^15^. The seismic profile crossed the extensional Songliao and Erlian Basins. The development of the Songliao Basin was characterized by early-stage rifting in the Early Cretaceous and late-stage sagging, and significant thermal subsidence persisted until the end of the Late Cretaceous^13^. The Erlian Basin is floored by volcanoes that primarily formed in the Upper Jurassic but may have continued into Lower Cretaceous strata, which is indicative of an active rifting regime in the basin^13^.

The NCC is an Archean craton that was stabilized during the Paleoproterozoic, and it was subsequently covered by a thick sequence of Proterozoic–Paleozoic sediments^16^. However, the NCC has become unstable since the Mesozoic and marked by crustal extension, lithospheric thinning with widely developed MCCs, extensional basins, regional granitic magmatism and large-scale gold mineralization^11^,^17^,^18^. The half-graben subsidence and large-scale thermal subsidence from the Mesozoic to the Cenozoic resulted in the formation of the Bohaiwan Basin^19^. The Tanlu Fault Zone is one of the largest continental faults on the East Asia continent, and it was initially an intra-continental transform fault zone that changed into huge normal faults that controlled the development of several graben or half-graben basins in the Early Cretaceous^20^.

## Results

Seismic data obtained from a dense seismic array of 60 temporary stations were used to image the crustal structures. The stations oriented in a SE-NW direction with a spacing of 10–17 km crossed the northern NCC and eastern CAOB over a distance of ~920 km from the China-North Korea border to the Sino-Mongolian border (Fig. 1). The stations were temporarily deployed from September 2007 to September 2008, and the observed profile is denoted as the NCISP-6.

**Figure 2:**
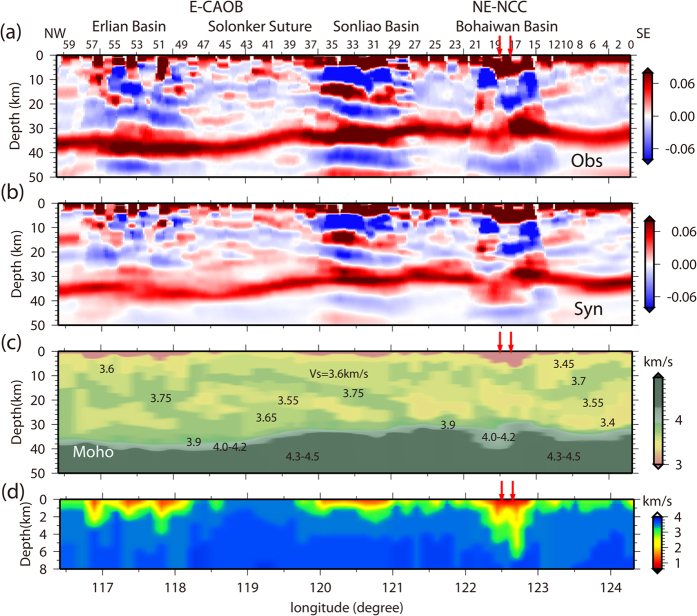
Synthetic CCP images compared with the observational CCP image, and the cross section of the crustal shear-wave velocity structure. (**a**) Observational CCP image calculated from the observed data; (**b**) CCP images calculated from the synthetic receiver functions; (**c**) shear-wave velocity structure of the crust and uppermost mantle compiled from the crustal models beneath each station; and (**d**) sedimentary structure. In the CCP images, red denotes the positive amplitude of the receiver function as annotated by the color bar, which indicates that velocity increases with depth. Blue denotes negative amplitudes and velocity decreases with depth. Certain station numbers are labeled on the top of the plot. Red arrows mark the surface site of the Tanlu Fault Zone.

We developed a velocity structure imaging technique for receiver functions to infer the crustal structures beneath the NCISP-6 from the teleseismic waveform records. Taking advantage of the concentration of energy from the P–S converted phases, direct imaging of the velocity discontinuity structures was achieved by stacking the amplitudes of the receiver functions in the depth domain using common conversion point (CCP) image stacking^21^,^22^. We simulated the observation CCP image from the data using synthetic CCP images to identify attributes of the velocity discontinuities in the CCP stacking section. The identified velocity discontinuities and corresponding depths were used as the constraint conditions in the global inversion of the receiver function waveforms, and crustal velocity models were obtained beneath each station. Figure 2a shows the CCP depth image beneath the NCISP-6 based on the data. Figure 2b shows the best-fitting CCP image (synthetic CCP image) stacked from synthetic receiver functions. A comparison between the synthetic CCP images and the observations indicates an accurate reconstruction of the crustal models. Traces of the intra-crust velocity discontinuities and the Moho interface for the Ps and PpPs phases are reproduced in the synthetic CCP image. Dual constraints from the CCP images and seismic waveforms can significantly enhance the reliability of receiver function imaging.

A shear-wave velocity image was compiled from the crustal models beneath each station obtained from the velocity structure imaging (Fig. 2c). A thin upper crust with a shear-wave velocity of 3.45–3.5 km/s is covered by a sedimentary sequence with a shear-wave velocity of 0.7–3.0 km/s (Fig. 2d) beneath the northern Bohaiwan (Xialiaohe) Basin (beneath stations 16–20), southern Songliao Basin (beneath stations 30–36), and eastern Erlian Basin (beneath stations 49–57). However, heterogeneous fabrics appear deep throughout the profile and are delineated by variable velocity discontinuities, where the velocity increases or decreases by approximately 3–8% at greater depths.

**Figure 3:**
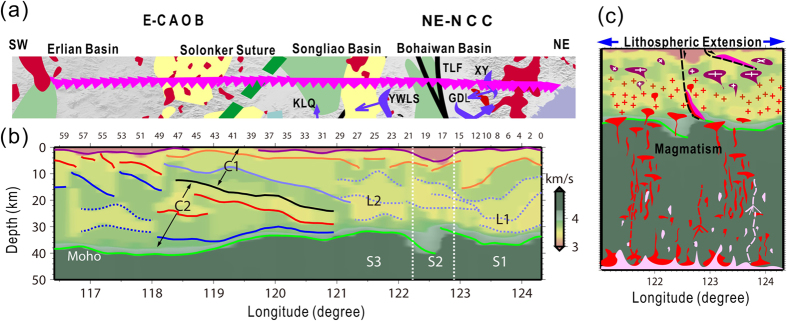
(**a**) Major tectonic units along with the location of the NCISP-6 profile. (**b**) The cross section of the shear-wave velocity structure beneath the NCISP-6 profile. The color lines mark the velocity discontinuities, and the dark line represents the boundary between C1 and C2. (**c**) Schematic illustration of the crustal rejuvenation by structural, magmatic and metamorphic processes in an extensional setting observed in the NCISP-6 profile.

The diffused and weakened PpPs phases from the Moho at certain stations (see Fig. S2 in the Supplementary material) indicated that the Moho interface beneath the NCISP-6 is not always a sharp velocity discontinuity and presents gradual transitions in seismic velocity^23^, with the shear-wave velocity (V_S_) of 3.9 km/s of the lower crust increasing gradually to 4.2 km/s. We defined the depth of the lower boundary of the transition zone as the depth of the Moho. The crustal thickness (Moho depth) shows a good agreement with results of the previous Donggou-Dongwuqi Geoscience Transect^24^ (Fig. S5 in the Supplementary material), which very closely parallels the NCISP-6 profile and shows a thin crust with an approximate thickness of 31–35 km in the southeast that increases to an approximate thickness of 38–41 km in the northwest. The velocity image of the NCISP-6 profile shows four significant features (Figs 2c and 3b): (1) crustal structures characterized by dominant low-velocity zones and a velocity inversion in the southeast; (2) large-scale dipping strata in the northwest; (3) offset of the Moho beneath stations 17–19; and (4) well-partitioned matching of the crustal structures with surficial geologic observations (Fig. 3a,b).

In the eastern NCC (stations 0–29), low-velocity zones occupy most of the space in the depth range of approximately 10–30 km and present shear velocities of 3.4 km/s and 3.55 km/s and inversion velocity discontinuities. In section S2, the upper interface of the crust-mantle transition zone increases from 30.2 to 33.5 km/s, is offset by 3.3 km beneath the Tanlu Fault Zone, and presents a thick transition zone of ~10 km (Fig. 3b). Beneath the CAOB (stations 30–60), the velocity structures derived from the seismological imaging are characterized by superimposed stratified crusts (C1 and C2 areas in Fig. 3b) separated by a southeastward-dipping interface. A successive increase of interval velocities from 3.45 km/s to 3.6 km/s to 3.75 km/s occurs downward in the upper region (C1 area), and a successive increase of interval velocities from 3.55 km/s to 3.65–3.75 km/s to 3.9–4.2 km/s occurs downward in the lower region (C2 area). The southeastward-dipping interface extends from the upper crust to the Moho beneath stations 29–48, and the crust thickens to a depth of 41 km beneath station 51.

## Discussion

As shown in Fig. 3a,b, the crustal structures are closely related to the surface geology tectonics. In the NCC, a dominant low seismic wave velocity with velocity inversion implies a compositional change and reflects the effects of strong granitic magmatism during the Mesozoic. In the CAOB, a low-angle dipping interface in the crust is likely the result of the amalgamation of the NCC and CAOB in the Late Permian.

Our crustal image beneath the eastern CAOB corresponding to the northwestern region of the NCISP-6 (stations 30–60) is characterized by superimposed stratified crusts (C1 and C2 areas in Fig. 3b) separated by a southeastward-dipping interface. We suggest that the crustal fabric indicates a southward subduction of the northern Paleo-Asian Ocean plate as suggested by geological observations^12^. Geological studies have suggested that the Late Mesozoic extension in the CAOB resulted from the post-orogenic gravitational collapse of the thickened crust following the Middle to Late Jurassic amalgamation of the North China-Mongolia block and the Siberian plate^13^,^25^. Our seismological observations indicated a crustal structure transformed by post-orogenic structural, deformational and magmatic processes in the eastern CAOB, whereas obvious rejuvenation of the continental crust was not observed.

However, the crustal structural characteristics in the eastern NCC, which are indicated in the southeastern part of the NCISP-6 profile, include a dominant low seismic wave velocity and velocity inversions in the L1 and L2 areas (Fig. 3b), thus indicating the evolution of crustal components and structures. Current crustal structural fabrics are manifestly composed of the products of tectonic processes whose cumulative duration spans much of the NCC’s history, and detailed geochemical studies have shown that two stages of crustal generation in the eastern NCC are Neoarchean and Mesozoic in age, and they correspond to the formation of ancient nuclei and continental crustal growth of the NCC, respectively^18^,^26^. Mid-Archean lithotectonic assemblages occur in the eastern block of the NCC but were only observed in the limited area of the Liaoji mobile zone^27^ (Fig. 1) in our study region. The Paleoproterozoic lithotectonic assemblage occurred in the western block of the NCC at a considerable distance from our imaged region^27^. Mesozoic igneous rocks are widespread throughout the eastern NCC. The most influential structural, metamorphic and magmatic processes in the NCC occurred in the Late Mesozoic, especially in the Early Cretaceous^28^ after the late Paleozoic amalgamation of the NCC and CAOB in the north and the NCC and Yangtze craton in the south. The Nd isotopic data show that the lower crust beneath the NCC is approximately 0.5 Ga younger than the upper crust^26^. This rejuvenation of the lower crust resulted from the addition of mantle-derived magma in the late Mesozoic. Although the crustal structures possibly formed during cratonization of the eastern NCC at approximately 2.5 Ga, the geological and geochemical observations of significant continental crustal growth and replacement of ancient continental crust by relatively young crustal materials during the Late Mesozoic^14^ suggest the reduced likelihood of ancient crustal structures in the decratonized NCC. Our results suggest that the observed changes in the crustal components and structures in the eastern NCC occurred in the Late Mesozoic.

This finding that the primary changes in the crustal components in the eastern NCC after its cratonization occurred in the Late Mesozoic and resulted from the addition of mantle-derived magmas is supported by geochemical and Sr-Nd-Hf isotopic features. Mesozoic igneous rocks are widespread throughout the northeastern NCC (Fig. 1), and they primarily consist of I- and A-type granitoids with minor mafic rocks and alkaline rocks. Whole-rock geochemical data, Sr-Nd isotopes, and *in-situ* zircon Hf isotopes indicate multiple sources (i.e., ancient and juvenile crustal materials) for the Mesozoic granitoids along with additional depleted mantle materials that changed the crustal components of the eastern NCC. This finding is confirmed by the identification of Mesozoic depleted mantle-derived mafic rocks in the eastern NCC^29^,^30^. Mantle-derived mafic magmas entered the continental crust in an extensional setting and triggered the anatexis of the lower crust materials, which generated felsic magmas. The mixing of mafic magmas and/or their fractionated products with crustal-derived felsic melts would produce hybrid magmas with various components and seismic velocities. These magmas would rise to various levels in the crust^31^,^32^,^33^, and the heavier melt residues would sink into the mantle. The thinner crust in the eastern part of the image transect is responsible for the delamination removal of heavier differentiated products in the magmas.

Both the addition of depleted mantle materials in the continental crust and the anatexis of the crustal materials changed the crustal components of the eastern NCC. In addition to the components, the intensity, duration, and distribution area of the magmas and the crustal rheology may have been sensitive to the structural transformation. The widespread MCCs and extensional basins and the emplacement of A-type granites and alkaline rocks in the eastern NCC (Fig. 1) indicate strong crustal extension and a weakened lower crust during the Late Mesozoic in the eastern NCC. The relatively shallow Moho discontinuity and the low seismic wave velocity, which was indicated by our seismological observations, suggest a weakened low-viscosity middle-lower crust with a high thermal gradient. The rheology of the middle-lower crust is consistent with the development of MCCs and domes, i.e., the Xiuyan magmatic dome, the Gudaoling MCC, the Yiwulushan MCC, and the Kalaqin magmatic dome (Figs 1 and 3a), along the NCISP-6 profile for the surface of sections S1 (stations 00–15) and S3 (stations 21–29). In the S2 section, an offset along the Moho occurs beneath the Tanlu Fault Zone, indicating an extensional fault. Geological observations show that the Tanlu Fault changed from a transformed fault into a normal fault in the Early Cretaceous^20^. Moreover, our seismic image reveals that intense faulting extended deep into the lithospheric mantle. The widespread melting and attendant rheological weakening of the lower crust may have facilitated the extension of the deep crust, and the extension of the shallow crust may have facilitated decompression melting.

A tectonic model was created in this study by combining the concepts of compositional change, crustal weakening and extension, and it was used to interpret the imaged crustal structure beneath the eastern NCC (Fig. 3b) as shown in Fig. 3c. In Fig. 3c, the lower velocity in the lower crust is a result of the granitic intrusion and delamination of the residual crustal material. Thus, we suggest that the crustal structural fabrics obtained by our seismological imaging recorded the results of crustal rejuvenation from the Late Mesozoic structural and magmatic processes in an extensional setting in the eastern NCC. The presence of melt and/or aqueous fluids in the crust that resulted in reduced seismic velocity may be an alternative tectonic interpretation of the dominant low seismic wave velocity structure of the eastern NCC. However, a thickened crust is not observed, such as in Tibet, and volcanic events have not occurred since the Pliocene. We argue that the probable interpretation is crustal rejuvenation from Late Mesozoic tectonism.

Continental rejuvenation involves diffuse lithospheric-scale deformations, and the behavior of the mantle lithosphere is likely the primary determinant of the type and duration of tectonic activity^2^. Previous studies based on xenoliths in Paleozoic kimberlites and Late Mesozoic to Cenozoic basalts have suggested that 80–140 km of the ancient cratonic mantle lithosphere of the eastern NCC was replaced by younger, less refractory lithospheric mantle during the Late Mesozoic^34^,^35^,^36^,^37^. The long-term duration of magmatism and previous geological, geophysical, and geochemical observations indicate that this process may be related to the subduction of the Paleo-Pacific Plate^11^. Thus, the subduction of the Paleo-Pacific Plate facilitated fluid/melt metasomatism, extensive melting and magmatism, and lithospheric extension and induced unsteady mantle flows underneath the eastern NCC. All of these processes would have rejuvenated the overlying continental lithosphere and subsequently resulted in the decratonization of the eastern NCC in the Early Cretaceous^11^,^38^. The fossil structural fabrics along the convergent boundary in the eastern CAOB have been preserved, which is indicated by our seismological observations, suggesting that the back-arc action of the Paleo-Pacific Plate subduction did not reach the hinterland of Asia.

Our seismological imaging shows the detailed crustal structure beneath northeastern China and provides insight into the rejuvenation processes of evolving crusts caused by structural, magmatic and metamorphic processes in an extensional setting in continental margins adjacent to oceanic plates. If our model accurately describes the continental rejuvenation of the eastern NCC, then similar processes may have led to the evolution of other continental blocks.

## Methods

A velocity structure imaging technique for receiver functions was used to construct the crustal velocity structure of the NCISP-6. The receiver functions were calculated using a time-domain maximum entropy deconvolution method. Three-component seismograms were selected from 122 teleseismic earthquakes with body wave magnitudes ≥ 6.0 and epicenter distances between 30° and 90°. For most of the stations, more than 60 receiver functions with high signal-to-noise ratios were selected after careful visual inspection, thus resulting in a total of 4366 receiver functions available for further analysis. The station locations and the number of receiver functions for each station are listed in Table S1 in the Supplementary material.

We confirmed the velocity structure of the NCISP6 based on the optimal match between a synthetic CCP image and an observational CCP image (Fig. 2a,b), and this process included four steps: 1) the observational CCP images were produced using the observed data and a regional average velocity model obtained from a previous study^39^; 2) the velocity models were inferred by a waveform inversion of stacked receiver functions for each station, and the interface depth from the observational CCP image applied as a constraint; 3) the synthetic CCP images were calculated based on the synthetic receiver functions with the same ray paths as the observations and inversed velocity models, and an improved observation CCP image was produced with the inversed velocity models; and 4) the observed and synthetic CCP images were compared to distinguish the real discontinuities from the multiple phases, the inversion space of the velocity models of each station were systematically adjusted and steps 2), 3) and 4) were executed repeatedly.

Introducing the synthetic CCP image allowed us to infer the overall seismic features, and the velocity interfaces in the CCP image established an intrinsic connection with the structure between adjacent stations. We compared the observational CCP image with the synthetic results from shallow to deep layers and examined each individual layer to distinguish the multiple PpPs and PsPs + PpSs waves generated by the shallow structures and identify the actual velocity discontinuities. The numerical examples of the synthetic test of the CCP images are provided in section C of the Supplementary material. The waveform inversion was performed using a nonlinear hybrid global inversion method^40^,^41^. In the inversion, a search was executed in the model parameter space to minimize the objective function (see the Supplementary material). We defined the a priori layered structure and parameterized the velocity model with the shear-wave velocity and layer thickness according to the high sensitivity of the receiver functions to these two factors. The P-wave velocity was estimated using the regional average Vp/Vs ratio obtained from previous studies. Because of the obvious differences among the direct P phase, the Moho Ps phase and other phases, an inversion was conducted to provide a detailed estimation of the objective function by adding different weight values to different parts of the stacked receiver function.

As shown in Fig. 2a,b, the traces of the intra-crust velocity discontinuities and Moho interfaces for the Ps and PpPs phases were reproduced in the synthetic CCP image. The excellent match between the synthetic and observation CCP images indicates an accurate reconstruction of the crustal structures. The final best-fitting shear-wave velocity models for all 60 stations are shown in Fig. 2c,d and section B of the Supplementary material. A high level of consistency was observed between the synthetic and observed waveforms for most stations. The velocity models of the sedimentary cover were compared with the geological explanations from oil fields near the NCISP-6 profile (Fig. S5d, in the Supplementary material), and the consistency between the sedimentary structure image and the geological profile supports the reliability of our receiver function imaging results for the basin area. The poor site conditions of stations 50, 51, 53, and 55, which have strongly localized heterogeneous shallow structures, resulted in extremely complex waveforms that are difficult to explain. Therefore, we did not interpret the imaging results of these stations in this study.

The receiver function imaging technique used in this study involves a synthetic test of the CCP image and waveform inversion. A reliability analysis was performed for the depths of the major interfaces and waveforms of the receiver function. The observational depth was measured by the local maximum amplitude of the observational CCP image. The synthetic depth was obtained from the waveform inversion. The depth errors at a 90% confidence interval were less than 1.4 km within the crust and less than 0.8 km along the upper boundary of the crust-mantle transition zone (Table S2 in the Supplementary material). The standard derivations of the interface depths were 0.38 km along the upper boundary of the crust-mantle transition zone and 0.48 km to 0.71 km within the crust. In the global inversion, we measured the degree of fitness of waveforms and amplitudes between the synthetic and observed data, with fitness determined by the objective function. The average values of the objective function of 60 stations decreased from 0.11 in the initial model to 0.080 in the final model (Table S3 in the Supplementary material), and these values were calculated in a time window of 0–6 s, starting at the arrival of the direct P wave and ending after the Ps phases from the Moho. The obvious reduction of the objection function values from 0.46 in the initial model to 0.22 in the final model (Table S3 in the Supplementary material) within a time window of 1–6 s without the inclusion of direct waves indicates that the coda behind the direct wave fits well. The close interface depths derived from the synthetic and observed CCP images and the consistency between the synthetic and observed receiver function waveforms confirm the reliability of our results.

## Additional Information

**How to cite this article**: Zheng, T.-Y. *et al.* Seismological constraints on the crustal structures generated by continental rejuvenation in northeastern China. *Sci. Rep.* **5**, 14995; doi: 10.1038/srep14995 (2015).

## Supplementary Material

Supplementary Information
