# Supplementary material for: Seismological constraints on the crustal structures generated by continental rejuvenation in northeastern China
Source: Sci Rep. 2015 Oct 7;5:14995. doi: 10.1038/srep14995 (PMC4595743; doi:10.1038/srep14995)
Supplement: Supplementary Information [file srep14995-s1.pdf]

Seismological constraints on the crustal structures generated by continental rejuvenation in northeastern China

Tian-Yu Zheng[1, *], Yu-Mei He[1], Jin-Hui Yang[2], Liang Zhao[2]
[1]Key Laboratory of Earth and Planetary Physics, Institute of Geology and Geophysics, Chinese Academy of Sciences, Beijing, 100029, China
[2]State Key Laboratory of Lithospheric Evolution, Institute of Geology and Geophysics, Chinese Academy of Sciences, Beijing, 100029, China

Supplementary: Seismic array, waveform inversion, synthetic test of the CCP images, and reliability analysis of the receiver functions imaging

A. Seismic array

Seismic data obtained from a dense seismic array of 60 temporary stations were used to image the crustal structure. The stations, oriented in the SE-NW direction with an average spacing of 10-17 km, crossed the northern NCC and eastern CAOB over a distance of ~920 km. The station locations and numbers of the receiver functions for each station are listed in Table S1.

Table S1 Seismic stations used for velocity structure imaging and the number of receiver functions selected for each station.

Station	Longitude (N)	Latitude (E)	Number of receiver functions
00	39 º59' 47.55"	124 º19' 14.26	57
01	40 º04' 45.48"	124 º13' 05.32	44
02	40 º09' 01.19"	124 º07' 32.52	70
03	40 º12' 59.36"	124 º02' 27.85	50
04	40 º17' 15.99	123 º57' 23.89	76
05	40 º22' 11.16	123 º51' 31.49	59
06	40 º26' 24.92	123 º48' 11.38	69
07	40 º30' 54.21	123 º41' 58.33	65
08	40 º34' 51.77	123 º35' 56.35	63
09	40 º40' 05.49	123 º31' 27.55	95
10	40 º44' 20.47	123 º26' 03.37	75
11	40 º48' 56.16	123 º20' 35.28	42
12	40 º53' 08.64	123 º15' 46.63	74
14	40 º59' 45.35	123 º05' 43.83	53
15	41 º03' 04.22	123 º01' 30.80	56
16	41 º12' 36.94	122 º50' 10.92	74
17	41 º19' 14.25	122 º41' 52.08	74
18	41 º25' 56.55	122 º34' 51.74	79
19	41 º31' 32.74	122 º25' 09.64	84
20	41 º38' 06.75	122 º18' 59.16	104

21	41 °46' 21.52	122 °10' 00.30	54
22	41 °53' 30.72	121 °59' 11.76	88
23	41 °59' 49.14	121 °51' 07.20	70
24	42 °08' 31.32	121 °41' 57.46	95
25	42 °12' 36.22	121 °37' 16.12	76
26	42 °18' 35.16	121 °28' 25.62	88
27	42 °23' 32.31	121 °21' 04.71	70
28	42 °30' 54.37	121 °10' 16.46	41
29	42 °38' 54.68	121 °03' 03.19	64
30	42 °43' 57.26	120 °52' 57.68	92
31	42 °49' 21.73	120 °46' 15.99	90
32	42 °58' 14.27	120 °39' 25.14	99
33	43 °02' 56.46	120 °32' 11.56	116
34	43 °07' 12.85	120 °23' 27.27	84
35	43 °16' 05.82	120 °12' 12.94	75
36	43 °23' 34.55	120 °02' 34.32	72
37	43 °29' 37.87	119 °52' 39.60	63
38	43 °35' 56.37	119 °45' 39.78	67
39	43 °42' 25.74	119 °35' 55.46	82
40	43 °48' 14.62	119 °28' 44.62	69
41	43 °55' 34.86	119 °18' 41.55	70
42	44 °00' 50.61	119 °11' 00.31	62
43	44 °08' 54.90	119 °00' 46.38	84
44	44 °14' 53.29	118 °53' 18.69	79
45	44 °21' 22.89	118 °40' 55.98	53
46	44 °28' 20.66	118 °32' 01.72	67
47	44 °34' 09.84	118 °25' 43.16	91
48	44 °40' 36.78	118 °15' 05.70	33
49	44 °46' 12.79	118 °06' 50.34	87
50	44 °51' 47.23	117 °57' 32.98	84
51	44 °56' 39.42	117 °49' 19.26	79
52	45 °03' 48.72	117 °37' 20.45	80
53	45 °11' 16.75	117 °32' 53.74	54
54	45 °15' 17.80	117 °21' 44.54	76
55	45 °22' 50.48	117 °09' 59.88	78
56	45 °29' 57.99	117 °05' 33.57	54
57	45 °35' 36.41	116 °52' 58.62	68
58	45 °40' 42.58	116 °42' 35.26	89
59	45 °47' 57.21	116 °33' 11.38	80
60	45 °52' 39.31	116 °24' 31.20	80

B. Results of waveform inversion

The velocity models used to calculate synthetic receiver functions were obtained by waveform inversion. The final best-fitting shear wave velocity models for all 60 stations are shown in Figure S1. The corresponding synthetic receiver functions calculated from the inverted velocity models for each station are also shown

superposed upon the data in which the excellent match between the synthetic and observed waveforms was displayed for most of stations.

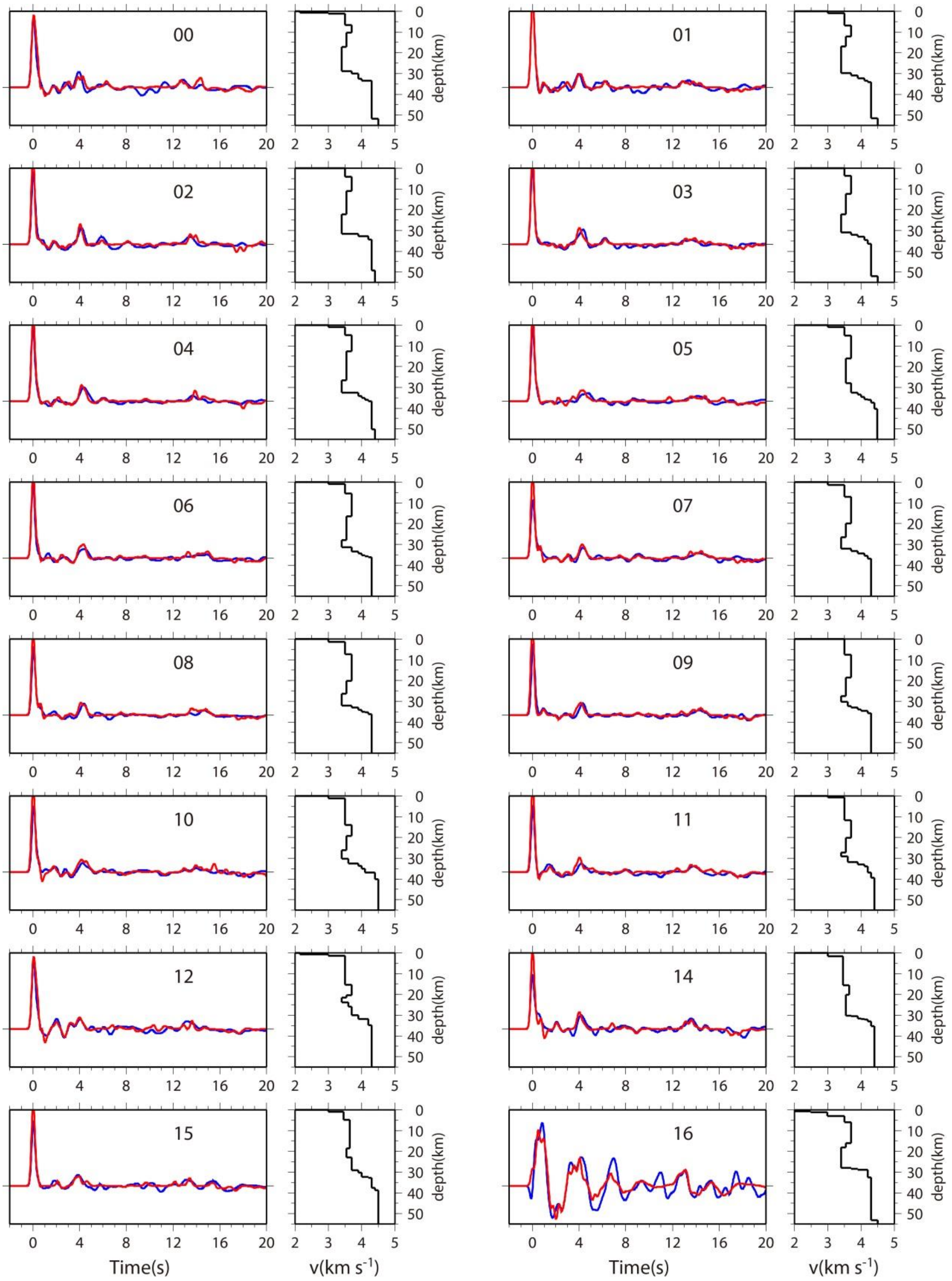

Figure S1 Shear-wave velocity models and comparisons between synthetics and observed receiver functions for all the 60 stations. For each station the best-fitting shear-wave velocity model is plotted in the right panel, the receiver functions are

plotted in the left panel with data in blue line and synthetic in red line. The station numbers are marked in the left panels.

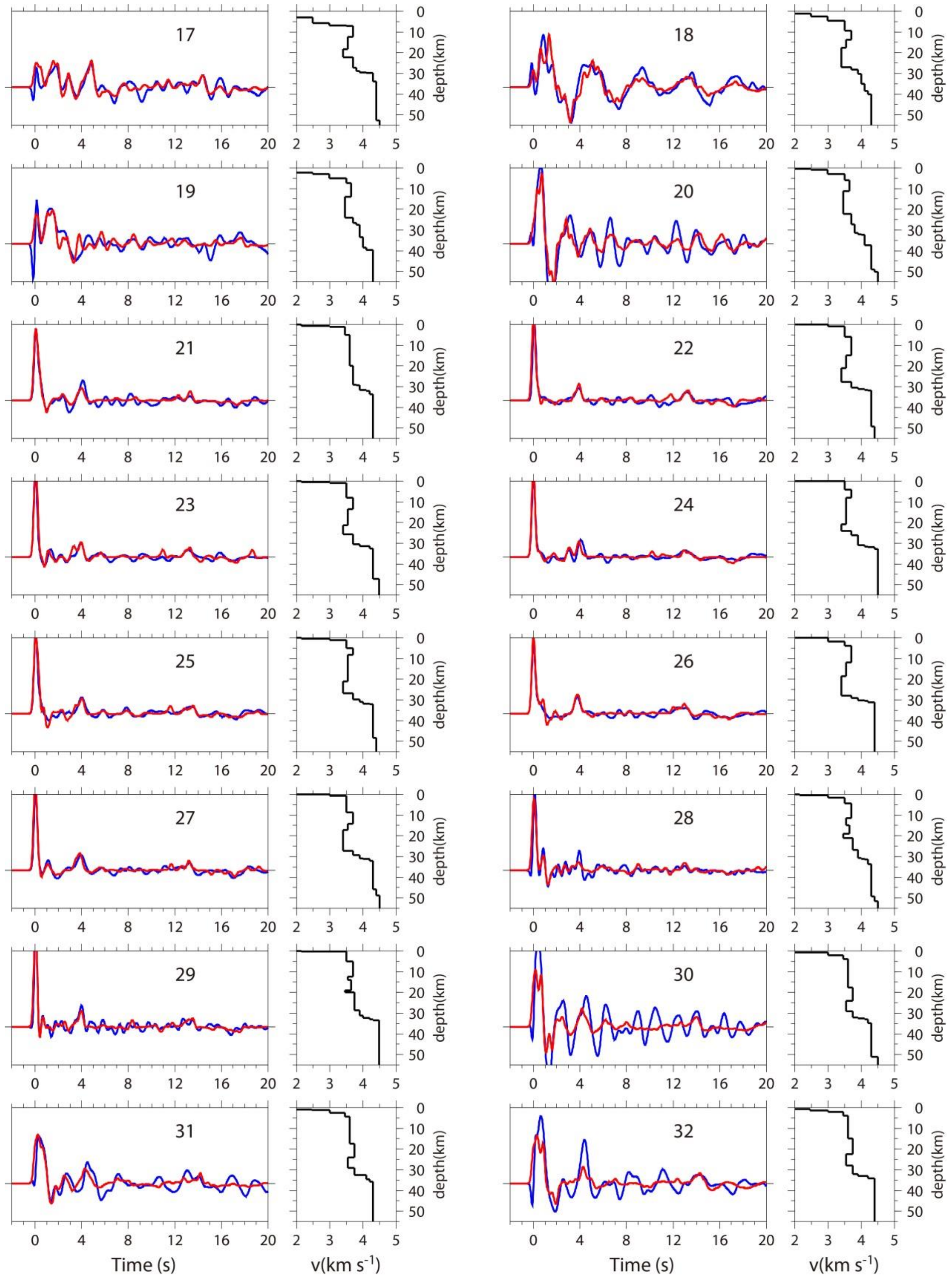

Figure S1 (continued)

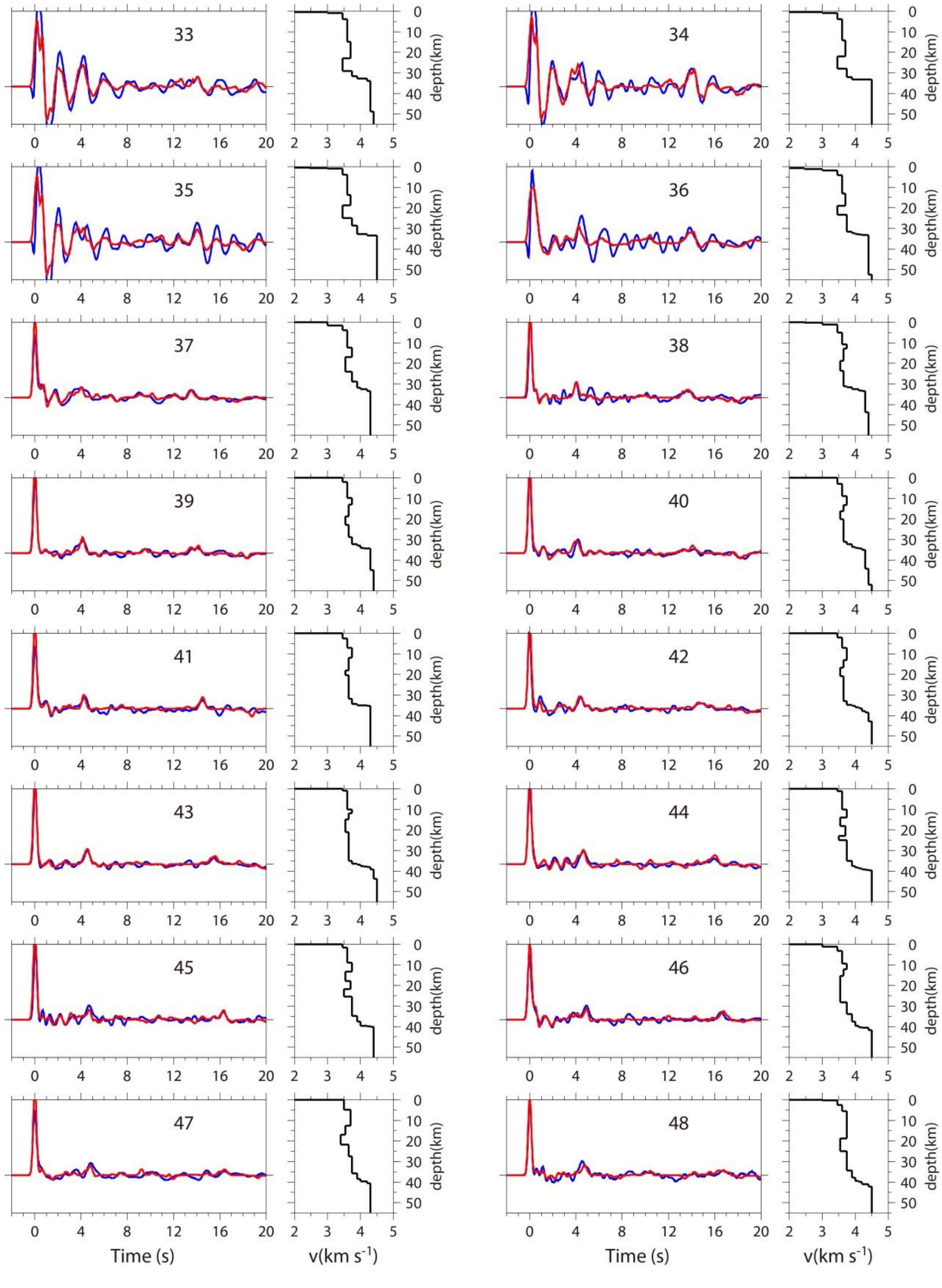

Figure S1 (continued)

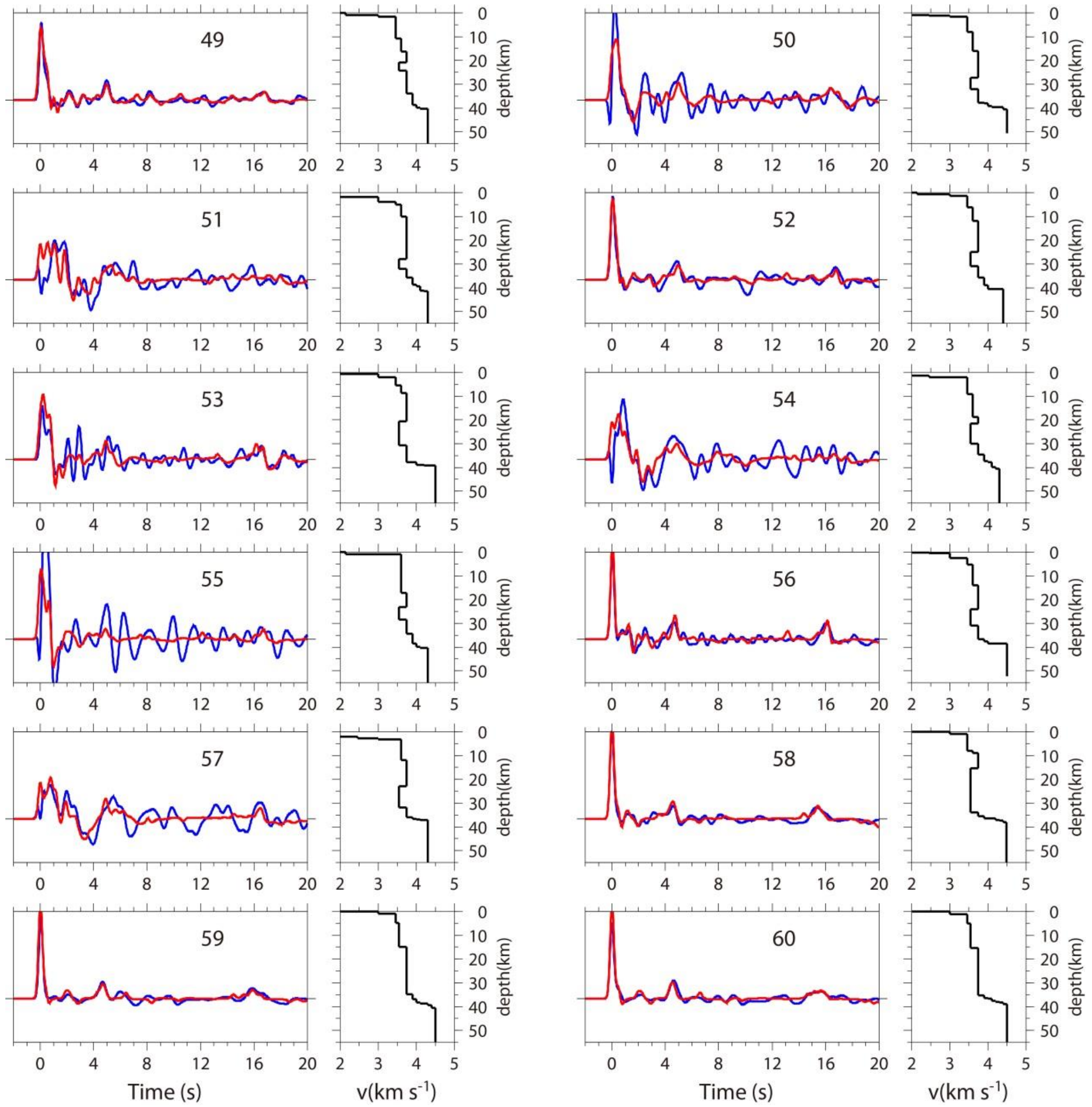

Figure S1 (continued)

We ranked the stacked receiver functions from data and the synthetics along the profile as shown in Figure S2 and compared their waveform variations. The waveforms of the synthetic receiver functions are in excellent agreement with the data. Major structural characteristics could be coherently detected beneath most of the stations. The velocity models of stations 17 and 19, which are located at both sides of the Tanlu Fault, are shown in Figure S3 for comparing.

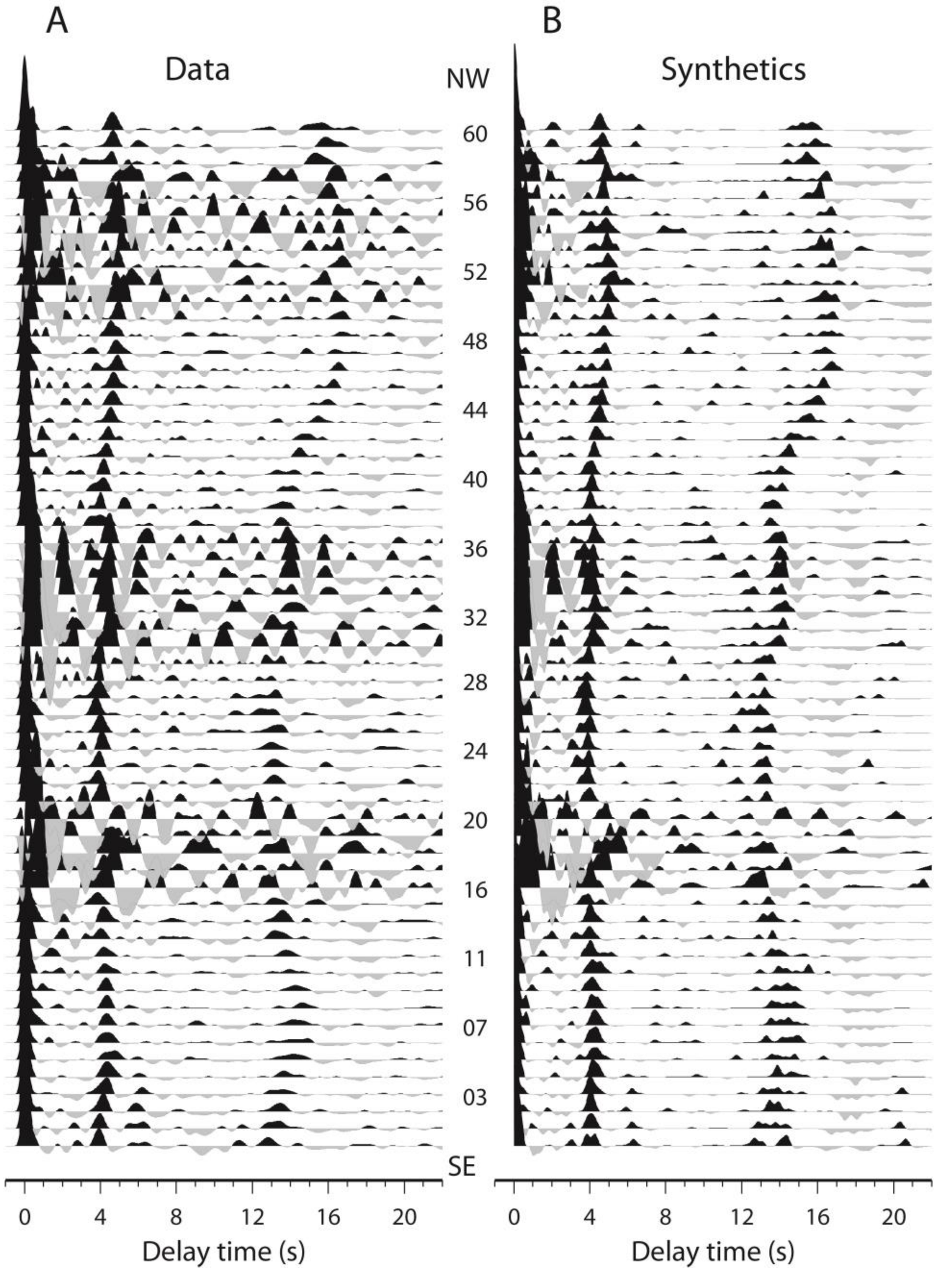

Figure S2. Cross-sections (from southeast to northwest) of P-receiver functions from (A) data by stacking and (B) synthetics. Traces are plotted in a time-window between -1 to 22 s with time-zero aligning with the onset of the P wave. Amplitudes of the traces are normalized by the maximum amplitude of all the traces. Some station numbers are labeled in the middle of the plots for ease of reference.

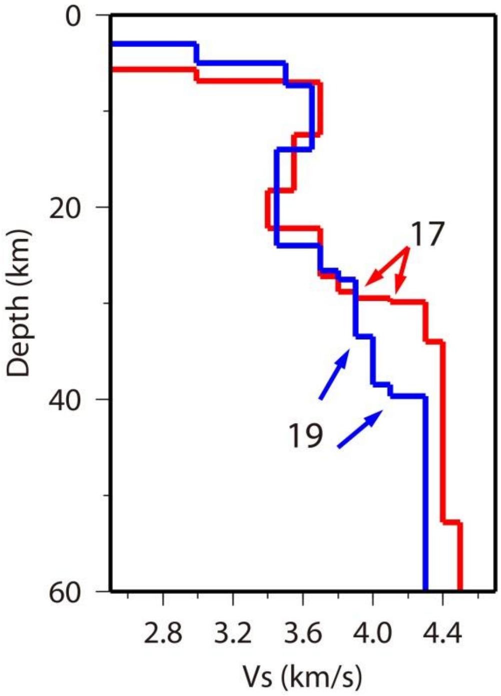

Figure S3. The velocity models of stations 17 and 19. The arrows indicate upper and lower boundaries of the crust-mantle transition zone.

C. Synthetic tests of the CCP images

The uncertainty in detecting a seismic discontinuity within the crust primarily arises from disturbances in the sedimentary cover and upper crust. We implemented a series of synthetic tests to determine whether a seismic signal in the CCP image represents an artifact or an actual velocity discontinuity. For example, when observing the effects of multiples from the sedimentary cover, we calculated the synthetic receiver functions by constructing models with a lower interface for the upper crust, deepening to 45 km beneath the inverted sedimentary cover models. As shown in Figure S4b, we can find the larger amplitudes of converted waves from the surface to a depth of more than 10 km, even though the sedimentary cover spans depths of 1-6 km. Obviously, the singles below the sedimentary cover, which are generated by the multiples, cannot be identified as velocity discontinuities. In observing the effects of the multiples from the upper crustal interface, we calculated the synthetic receiver functions by constructing models with a lower interface for the middle crust, deepening to 45 km beneath the inverted upper crustal models, and produced synthetic CCP image (Fig. S4c). Comparing the synthetic CCP image with the observation CCP image (Fig. S4a), we can clearly see that signals from multiples appeared throughout the entire crust. Hence, the signals that appeared in the observation CCP image but not in the synthetic CCP image should be considered as the crustal interfaces. The structural framework of the waveform inversion was then iteratively adjusted following the synthetic CCP test.

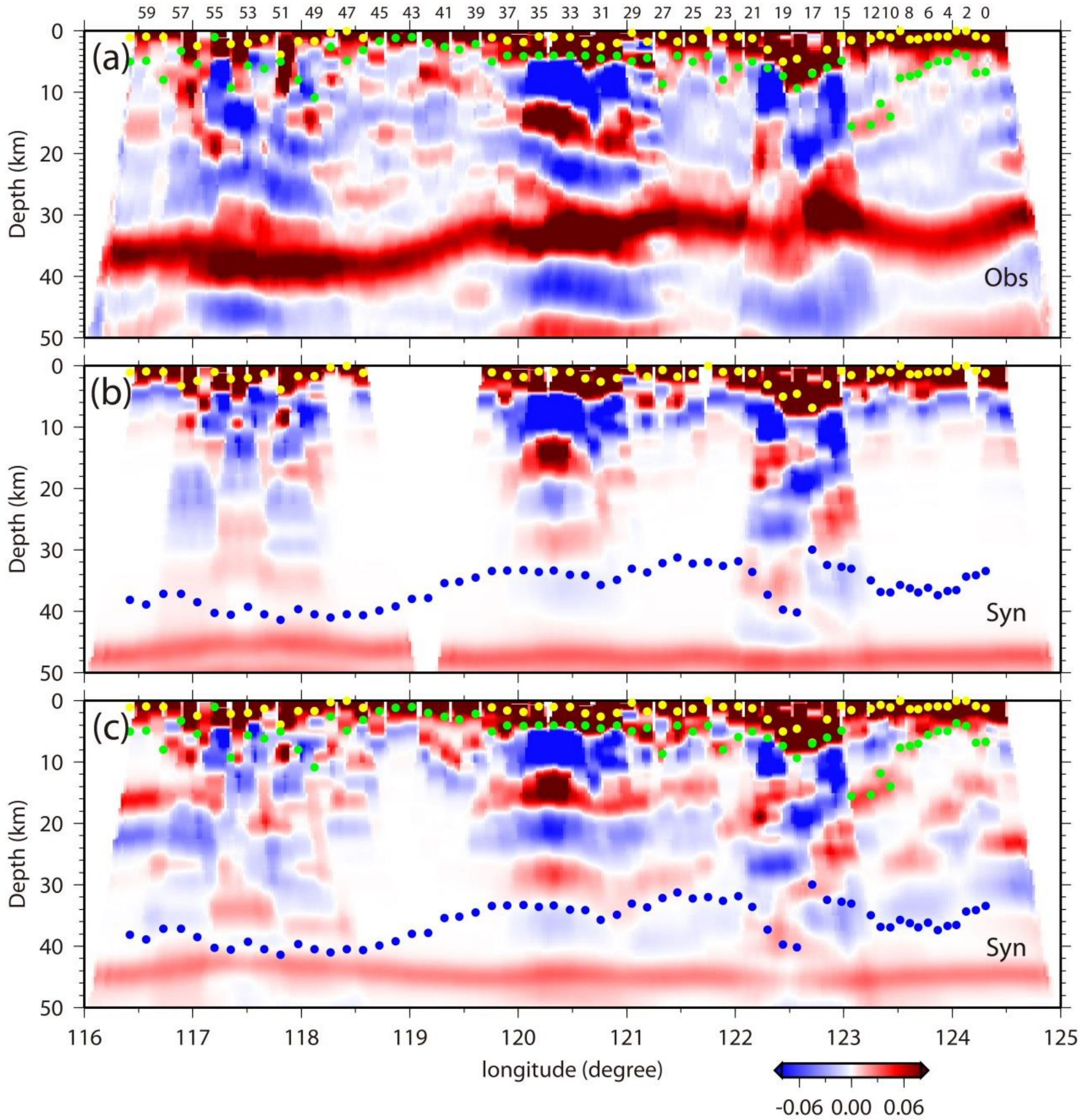

Figure S4. Synthetic CCP images of the crust along the NCISP-6 profile compared with observation CCP image. (a) Observation CCP image calculated from data; (b) synthetic CCP image calculated from the synthetic receiver functions based on the velocity models with a lower-interface of the upper-crust deepen to 45 km beneath inverted sedimentary model. (c) CCP image calculated from the synthetic receiver functions based on the velocity models with a lower-interface of the middle-crust of 45 km beneath inverted upper-crust models. Annotation of receiver function amplitude is the same as that in the caption of Figure 2. Dots in the CCP image mark velocity discontinuities in the best-fitting models, including basement (yellow), bottom interface of upper crust (green), and Moho (blue). Certain station numbers are labelled on the top of the plot.

Because the stacking profile in depth domain is constructed by time-to-depth conversion based on a defined velocity model, it is necessary to carefully assess the

ability of stacking-based CCP imaging to constrain the depths of discontinuities in a region with thick sedimentary cover. Figure S5a shows the CCP depth image constructed using an average crustal model for the NCC, in which no sedimentary structure is included. The resultant CCP image (Fig. S5b) was constructed using the imaging models. As shown in Figure S5a, the depth distributions of Moho (lower boundary of the crust-mantle transition zone, blue line) beneath the Bohaiwan (Xialiaohe) basin and the Sonliao basin are apparently deeper than our seismic imaging result (green line). In Figure S5c, we compared the Moho image with the previous estimation from seismic refraction observation of the Donggou-Dongwuqi Geoscience Transect[1] (red dashed line). In Figure S5d, the geologic sections from oil fields near the NCISP-6 profile[2-4] are superposed on our velocity profile. The coincidence of sedimentary structure image and geological profile verifies the reliability of our receiver function imaging results in the basin areas.

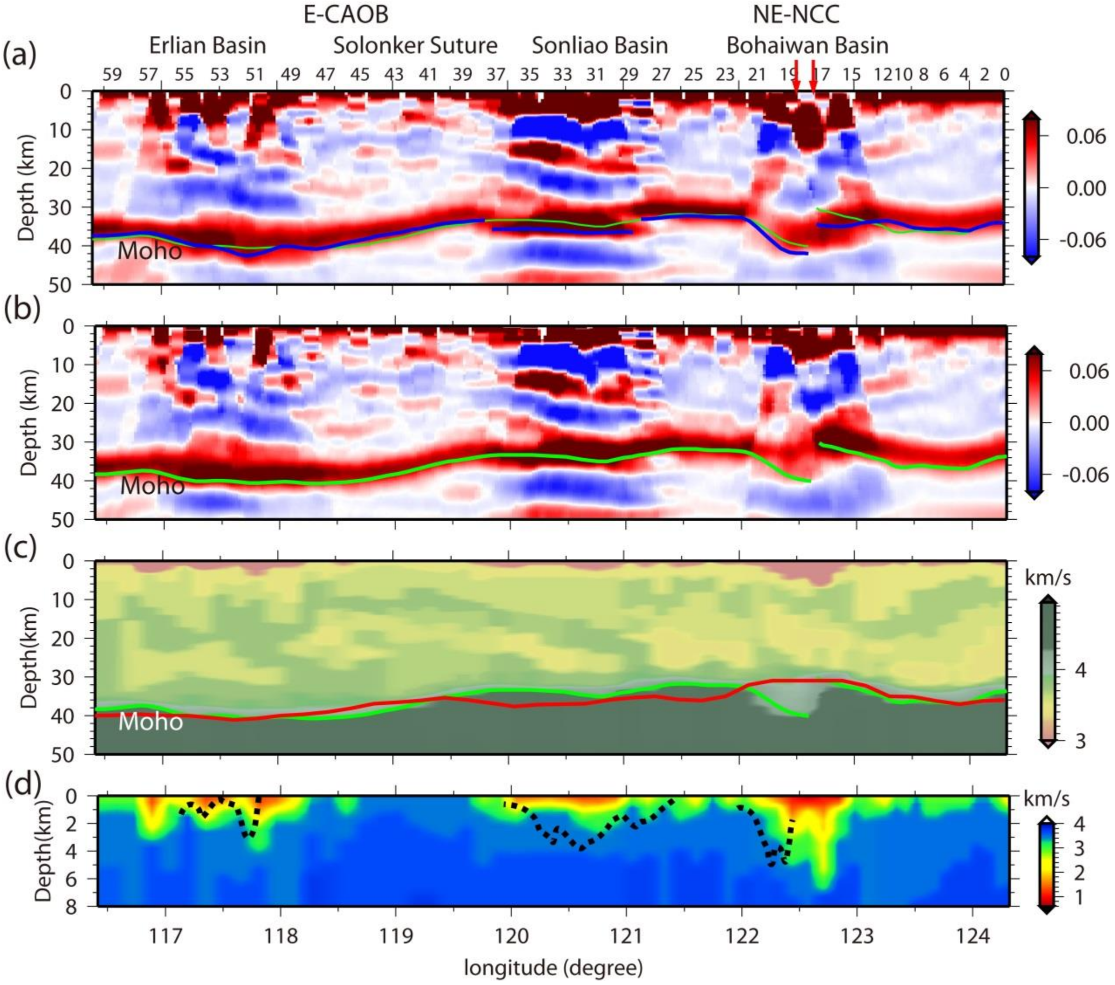

Figure S5. CCP images based on different velocity models and comparison of the structures beneath the basins. (a) CCP images constructed using an average crustal model for the NCC, in which no sedimentary structure is included; (b) CCP image constructed using the resultant velocity models; (c) shear-wave velocity structure with superposed Moho image from seismic refraction observations of the Donggou-Dongwuqi Geoscience Transect (red line); and (d) sedimentary structure with

superposed geological explanations (black dashed line) from oil fields near the NCISP-6 profile. The color lines mark the Moho discontinuities (bottom interface of the crust-mantle transition zone), in which red line marks the Moho from the Donggou-Dongwuqi Geoscience Transect, green and blue lines mark the Moho from the CCP images in (a) and (b), respectively. Certain station numbers are labeled on the top of the plot. Red arrows mark surface site of the Tanlu Fault Zone.

D. Reliability analysis of the receiver function imaging

The receiver function imaging technique used in this study involves the synthetic test of CCP images and waveform inversion. The reliability analysis was carried out for the depths of major interfaces and the waveforms of receiver function. The interface depths of each station are listed in the Table S2, in which the observation depths were measured by the local maximum amplitudes of observation CCP image, and the synthetic depths were obtained from waveform inversion. The depth errors at a 90% confidence interval were less than 0.78 km along the upper boundary of the crust-mantle transition zone, and less than 1.4 km within the crust. The standard derivations of the interface depths were 0.38 km along the upper boundary of the crust-mantle transition zone, 0.48 km and 0.71 km within the crust, respectively.

In the global inversion, the best-fitting between the synthetic receiver function Y(t) and observed one O(t) is searched by minimizing the objective function[5-6]

$$\mathrm{OBJ} = 1 - \frac{2\sum_t O(t)\times Y(t)}{\sum_t O^2 + \sum_t Y^2(t)}.$$

This OBJ measures the degree of fitness of waveforms and amplitudes between O(t) and Y(t). The OBJ of each station are listed in the Table S3. We calculated the OBJ in a time windows of 0-6 s and 1-6 s respectively, in which the onset of the P wave is 0 s, and the arrival times of the Ps phase from the Moho are less than 6 s for all the stations. The average values of the objective function of 60 stations reduce from 0.11 in the initial model to 0.080 in the final model within a time window of 0-6 s. The average values of the objective function of 60 stations reduce from 0.46 in the initial model to 0.22 in the final model within a window of 1-6 s. The obvious reduction of objective function values from the initial model to the final model indicates that the coda behind the direct wave fits well.

Table S2. Comparison of interface depths between observation and synthetic CCP images

Sta-tion	Depth of upper boundary of crust-mantle transition zone (km)		Depth of upper boundary of low-velocity zone (km)		Depth of dipping interface (km)	
	Obs.	Syn.	Obs.	Syn.	Obs.	Syn.
00	32.4	32.6	10.4	10.4		

01	32.7	32.2	11.2	12.2		
02	33.3	32.8	11.2	11.1		
03	33.5	33.0	11.8	12.4		
04	33.9	34.2	12.7	12.6		
05	34.4	34.1	14.9	15.9		
06	35.0	34.5	17.4	16.6		
07	34.3	34.4	18.2	19.8		
08	34.0	34.1	18.7	20.1		
09	33.8	33.9	18.7	18.5		
10	33.6	33.7	19.5	19.3		
11	33.3	32.9	19.6	20.3		
12	32.4	31.9	20.0	20.5		
14	31.1	31.6	20.0	20.2		
15	30.3	30.3	18.1	18.5		
16	29.8	28.8	17.1	16.0		
17	29.8	29.5	11.2	12.5		
18	31.2	30.2	12.8	13.9		
19	32.9	33.5	11.2	14.0		
20	32.5	32.1	11.0	11.2		
21	32.3	31.5				
22	31.4	31.3	14.9	14.9		
23	31.8	31.4	13.2	13.5		
24	31.2	31.0	9.9	8.1		
25	30.7	30.9	9.2	8.3		
26	29.9	30.2	12.4	12.0		
27	30.6	30.9	13.9	14.2		
28	31.1	30.6	12.1	11.4		
29	31.9	31.3	11.6	12.5		
30	32.7	32.5			23.6	24.1
31	33.0	32.5			23.4	24.0
32	33.2	32.9			22.8	22.5
33	32.4	32.7			22.2	23.0
34	33.2	33.2			22.6	22.1
35	34.1	32.7			18.9	18.8
36	33.8	32.3			17.5	19.1
37	32.4	32.0			17.6	17.0
38	32.8	32.4			12.4	12.9
39	32.4	32.9			13.2	13.1
40	34.3	33.7			12.5	13.3
41	34.7	35.1			11.5	11.9
42	36.1	35.3			12.4	13.0
43	36.9	37.0			12.6	12.0
44	37.7	37.6			13.5	14.0
45	38.0	37.7			13.2	13.2
46	38.1	37.7			11.6	12.2
47	37.9	38.5			12.5	12.6
48	37.9	37.8				
49	38.5	38.7				

50	38.3	38.1				
51	38.3	38.6				
52	38.4	38.9				
53	37.4	37.8				
54	38.6	37.4				
55	38.7	38.4				
56	37.1	37.5				
57	36.1	36.6				
58	36.6	36.5				
59	36.4	36.9				
60	36.4	36.5				
SD	0.38		0.71		0.48	
CI	(-0.58, 0.78)		(-1.1, 1.4)		(-0.62, 0.75)	

SD: standard derivations; CI: (90% confidence interval)

Table S3. Objective functions of waveform inversion from the initial and the final models

Station	Time window for calculating Objective function			
	0-6 s		1-6 s	
	Initial model	Final model	Initial model	Final model
00	0.056	0.040	0.52	0.22
01	0.043	0.018	0.41	0.22
02	0.107	0.033	0.72	0.10
03	0.057	0.029	0.45	0.14
04	0.057	0.029	0.29	0.11
05	0.044	0.027	0.32	0.28
06	0..056	0.036	0.37	0.13
07	0.058	0.054	0.47	0.14
08	0.067	0.026	0.61	0.18
09	0.035	0.045	0.31	0.19
10	0.049	0.040	0.47	0.16
11	0.066	0.049	0.36	0.25
12	0.066	0.027	0.57	0.08
14	0.103	0.100	0.55	0.25
15	0.100	0.035	0.74	0.18
16	0.170	0.068	0.36	0.10
17	0.568	0.129	0.60	0.06
18	0.216	0.153	0.29	0.16
19	0.064	0.135	0.07	0.17
20	0.142	0.100	0.32	0.20
21	0.086	0.032	0.68	0.24
22	0.032	0.018	0.57	0.11

23	0.046	0.029	0.55	0.19
24	0.101	0.035	0.62	0.26
25	0.072	0.032	0.65	0.19
26	0.044	0.029	0.23	0.10
27	0.061	0.019	0.34	0.11
28	0.070	0.052	0.51	0.30
29	0.088	0.020	0.83	0.22
30	0.289	0.235	0.55	0.42
31	0.141	0.114	0.39	0.33
32	0.234	0.199	0.51	0.38
33	0.210	0.091	0.43	0.11
34	0.136	0.085	0.31	0.13
35	0.218	0.130	0.38	0.18
36	0.167	0.105	0.62	0.40
37	0.058	0.065	0.23	0.25
38	0.075	0.031	0.98	0.35
39	0.070	0.020	0.74	0.12
40	0.056	0.032	0.40	0.24
41	0.098	0.043	0.75	0.15
42	0.070	0.049	0.30	0.23
43	0.046	0.011	0.49	0.08
44	0.030	0.032	0.21	0.19
45	0.058	0.035	0.68	0.29
46	0.092	0.048	0.60	0.18
47	0.054	0.073	0.13	0.29
48	0.056	0.038	0.29	0.30
49	0.046	0.027	0.15	0.14
50	0.168	0.176	0.47	0.44
51	0.483	0.415	0.43	0.29
52	0.094	0.043	0.64	0.26
53	0.133	0.226	0.28	0.63
54	0.340	0.276	0.46	0.25
55	0.229	0.377	0.50	0.59
56	0.140	0.055	0.58	0.29
57	0.205	0.162	0.25	0.22
58	0.031	0.024	0.26	0.12
59	0.051	0.021	0.50	0.10
60	0.065	0.038	0.51	0.11
Aveage value	0.114	0.080	0.46	0.22

1. Lu, Z. X. & Xia, H. K. Geoscience Transect Map from Dong-Ujimoqinqi, Inermogolia to Donggou, Liaoning, China (1:1,000,000) and Explanatory Notes (in Chinese) (Seismological Publishing House, Beijing, China, 1992).
2. Meng, Q. R., Hu, J. M., Jin, J. Q., Zhang, Y. & Xu, D. F. Tectonics of the late Mesozoic wide extensional basin system in the China-Mongolia border region, *Basin Research*, **15,** 397–415, (2003).

3. Tong, H. M., Mi, R. S., Yu, T. C., Liu, B. H. & Yang, J. Y. The Strike-Slip Tectonics in the Western Liaohe Depression, Bohai Bay Basin. *ACTA Geologica Sinica*, **82,** 1017-1026, (2008), (in Chinese).
4. Cheng, J., et al. A probing of the cause mechanism in positive reverse structure, Daan fault, Changlin fault depression. *Petrochemical Industry in Inner Mongolia*, **15,** 1-4, (2014), (in Chinese).
5. Liu, P. C., Hartzell, S. & Stephenson, W. Non-linear multiparameter inversion using a hybrid global search algorithm, applications in reflection seismology. *Geophys. J. Int.*, **122,** 991-1000 (1995).
6. Ai, Y. S., Liu, P. C. & Zheng, T. Y. Adaptive hybrid global inversion algorithm. *Scientia Sinica (B)*, **41,** 137-143 (1998).
